# De-escalating axillary surgery in node-positive early breast cancer patients undergoing primary surgery: current evidence and recommendations

**DOI:** 10.1186/s43046-025-00318-7

**Published:** 2025-09-22

**Authors:** Hussain Abdulla, Eman Hamza, Maha Alsendi

**Affiliations:** 1 Government Hospitals, Manama, Bahrain; 2 Bahrain Oncology Center, Busaiteen, Bahrain

**Keywords:** Sentinel lymph node biopsy, Axillary lymph node dissection, Targeted axillary dissection, Clinically node-positive axilla, Upfront surgery, Primary surgery, De-escalation

## Abstract

Landmark trials have shown that axillary lymph node dissection (ALND) can be safely omitted in early breast cancer patients with 1–2 positive nodes. Despite lack of prospective data, the National Comprehensive Cancer Network (NCCN) guidelines recommend that patients with 1–2 suspicious or biopsy-proven positive lymph nodes having primary surgery can undergo sentinel lymph node biopsy (SLNB). In the era of de-escalation of axillary surgery, breast cancer management in patients with clinically node-positive (cN +) axilla is driven by tumour biology and response to neoadjuvant chemotherapy (NACT). In this review, we discuss the management of the axilla in early breast cancer patients with low-volume biopsy-proven nodal disease and summarise the evidence supporting omission of ALND in these patients undergoing primary surgery.

## Introduction

In the last few decades, de-escalation of axillary surgery has evolved from ALND to SLNB, and even omission of axillary surgery in select patients. The NSABP B-32 and Milan trials established the oncological safety of SLNB in clinically node-negative (cN0) patients [1, 2]. Other trials, including IBCSG 23–01, ACOSOG Z011, AMAROS, OTOASOR, SINODAR ONE and SENOMAC, showed no difference in axillary recurrence in patients with limited nodal involvement treated with SLNB alone compared to ALND [3–8]. Notably, patients with cN + disease were excluded in these trials, including those whose axillary lymph nodes are clinically not palpable but suspicious on axillary ultrasound, or even biopsy-proven to contain metastatic disease.

Physical examination alone was used to define cN0 status in ACOSOG Z011 [4]. The American Society of Clinical Oncology (ASCO) guideline recommends that preoperative axillary ultrasound is not mandatory for early-stage T1 and T2 breast cancer patients, in whom SLNB is likely to be negative [9]. In clinical practice, however, ultrasound is standard of care for patients with a new breast cancer diagnosis, to detect radiologically suspicious or pathologic nodes that are not always palpable [10]. Axillary ultrasound is important in human epidermal growth factor receptor 2 (HER2)-positive and triple-negative breast cancers (TNBC), since positive nodal status is an indication for NACT in these subtypes [11]. These patients have high pathological complete response (pCR) rates [12]. NACT improves survival in these patients, offers breast and axillary downstaging and allows for escalation of adjuvant therapy if residual disease is present [13].

For hormone receptor (HR)-positive and HER2-negative disease, the management of the axilla is not straightforward and varies by clinical stage and menopausal status [14]. Axillary management in these patients has become even more complex after the RxPONDER trial, which showed that postmenopausal women with 1–3 positive nodes and an Oncotype DX score of 0–25 do not benefit from chemotherapy [15]. This suggests that postmenopausal women with cN + disease should undergo primary surgery, traditionally an ALND, so that genomic assays can be used to guide chemotherapy decisions. This trial also reported that chemotherapy is associated with a survival benefit in younger patients with node-positive disease, suggesting a role for NACT in premenopausal women. However, this may not be optimal for these patients, as they have lower rates of nodal pCR, meaning that most of these patients will still need ALND [12].

The NCCN guidelines state that patients with 1–2 suspicious or biopsy-proven lymph nodes undergoing primary surgery can be treated with SLNB alone [11]. This guidance is extrapolated from the aforementioned studies, as well as the NSABP B-04 trial, which showed that ALND does not result in better survival even in patients with node-positive breast cancer [16]. This will minimise surgical overtreatment among patients with ‘low nodal burden’ that otherwise meet ACOSOG Z011 or AMAROS criteria for omission of ALND [10]. However, there is no prospective data to support this approach. Preoperative imaging lack accuracy in predicting the final axillary nodal burden and radiologists have difficulty quantifying the number of abnormal nodes on ultrasound [17, 18].

At our multidisciplinary team (MDT) meetings, we face difficulty differentiating between luminal HER2-negative tumours with ‘low’ and ‘high’ axillary nodal burden and whether such patients should undergo primary surgery or NACT. If these patients with cN + disease are candidates for primary surgery, there is another challenge of whether they are more appropriate for ALND or SLNB. The aim of this review is to summarise the evidence supporting the oncological safety of omission of ALND in patients with cN + disease undergoing primary surgery and to provide an algorithm for axillary management in these cases.

## HER2-positive and TNBC

NACT is widely adopted as standard of care for all patients with node-positive operable HER2-positive and TNBC, who have a higher chance of pCR rates, approximately 65% and 50%, respectively [9, 12]. A pCR, or low residual cancer burden, is associated with a favourable prognosis in these patients [13]. NACT enables in vivo drug sensitivity testing and gives information about adjuvant treatment in these subgroups, offering the opportunity for trastuzumab emtansine and capecitabine if residual disease is present [19]. More importantly, it allows downstaging and can increase breast conserving surgery (BCS) rates, especially when upfront mastectomy is recommended, and avoid ALND after NACT. In addition, patients with early-stage HER2-positive and TNBCs are at risk of nodal upstaging if they undergo primary surgery with SLNB [20, 21]. In Bahrain, about a quarter of early-stage cN0 patients with HER2-positive tumours have nodal metastasis after primary surgery with SLNB [22].

Upfront surgery may be appropriate for some patients with small T1 cancers. For instance, patients with pT1N0 HER2-positive tumours may benefit from a milder adjuvant treatment regimen limited to paclitaxel and trastuzumab only and those with pT1a TNBC may not require chemotherapy at all. Other patients should undergo preoperative axillary imaging by ultrasound and biopsy of any suspicious node is warranted to expand potential benefit from NACT. Even when 1–2 suspicious or biopsy-proven nodes are present, applying the NCCN guidelines by performing primary SLNB is not appropriate. We advocate that the NCCN guidelines be revised to exclude this cohort of patients from primary surgery with SLNB if only 1 or 2 suspicious or biopsy-proven lymph nodes are identified on axillary ultrasound, unless they have contraindications for NACT.

## HR-positive and HER2-negative disease

Axillary management in this cohort of patients with cN + disease is uniquely complex. Unlike patients with HER2-positive and TNBC, these patients have nodal pCR rates of approximately 20%, meaning that 80% will end up with ALND [23]. Therefore, the use of NACT for axillary downstaging alone should not be warranted in these patients. The use of Oncotype DX has been proposed to guide NACT decisions, particularly in postmenopausal patients with biopsy-proven disease [14]. However, studies have shown that Oncotype DX is not useful to predict benefit from NACT and should not be used to guide decisions regarding the extent of axillary surgery in these patients [24, 25]. In addition, results from the RxPONDER trial showed that Oncotype DX identifies postmenopausal patients with node-positive disease who do not benefit from chemotherapy, thereby making NACT a suboptimal choice for many of these patients [15]. With the results of this trial, more patients with cN + disease will undergo primary surgery rather than NACT [17]. However, whether primary surgery should be ALND or SLNB is a matter of debate.

## Omission of ALND in patients with low axillary burden undergoing primary surgery

Traditionally, ALND is the standard of care for patients with biopsy-proven nodal metastasis undergoing primary surgery [26]. Retrospective studies on patients with cN + disease have shown that approximately 50% of cases have only 1 or 2 positive nodes at primary surgery and could therefore be spared the morbidity of ALND [27–29]. Based on these data, the NCCN guidelines recommend that patients with 1–2 suspicious or biopsy-proven nodes can undergo SLNB [13]. However, data from these studies were from patients who underwent primary ALND. Given the large amount of research addressing oncological safety of SLNB in the neoadjuvant setting, the lack of prospective data for patients undergoing primary surgery is extremely striking.

Evidence to support this approach is from a retrospective National Cancer Database analysis of 12,560 patients with limited cN + T1-2 breast cancer undergoing either primary SLNB and regional nodal irradiation (RNI) or ALND (with or without RNI), which showed that overall survival was similar between those undergoing SLNB and ALND [30]. Nevertheless, the MUTAS trial showed SLNB was not reliable in patients with biopsy-proven nodes undergoing primary surgery, with a false-negative rate (FNR) of approximately 30% [31]. The SENOMAC trial included patients whose axillary nodes were not palpable on clinical examination but were suspicious on ultrasound or even biopsy-proven positive [8]. However, patients with suspicious lymph nodes on ultrasound accounted for 13% of the SLNB group in this trial, with only 1.3% being biopsy-proven as positive. Thus, caution is warranted to omit ALND in this patient population.

Clipping the biopsy-proven lymph node and its subsequent retrieval along with a SLNB, a targeted axillary dissection (TAD), decreases the FNR of SLNB in cN + patients after NACT [32]. The added benefit of TAD over SLNB on oncological outcomes has been questioned, with recent data showing that SLNB alone is comparable to TAD in terms of axillary recurrence [33]. However, TAD can be extrapolated for use in primary surgery in those with 1 or 2 suspicious nodes in order to ensure removal of the positive node and increase surgical precision. In a feasibility study of the TAXIS trial, the FNR of tailored axillary surgery (TAS), which includes selective removal of sentinel nodes, as well as palpably suspicious and targeted nodes, was only 2.4% and the clipped node was successfully retrieved in 96.4% of patients [34]. However, the long-term oncological outcomes will not be known until 2029. A retrospective single-institution experience from South Korea reported that 10-year oncological outcomes of TAS were not inferior to those with ALND, but the incidence of lymphoedema was significantly higher in the ALND group [35]. Nevertheless, this study is limited by lack of information about the success of retrieving the targeted node. Although evidence from literature suggests that TAD may be feasible and a safer approach in the primary surgery setting, robust prospective data is needed [26].

The NCCN guidelines, which allow SLNB in those with 1–2 biopsy-proven positive nodes, still recommend completion ALND in patients who have more than 2 positive nodes [11]. This means that the vast majority of patients would still require ALND [17]. Another study evaluating TAD for cN + patients undergoing primary surgery is TADEN, which will also assess if there is a reduction in recommendations for completion ALND, such as omission of ALND if 3 positive nodes are identified in the setting of axillary radiation [26]. Similarly, feasibility of SLNB was evaluated in a prospective single-arm study at Memorial Sloan Kettering Cancer Center, which showed, among women with HR-positive/HER2-negative cancers with palpable biopsy-proven axillary metastases and no more than 3 abnormal nodes on US, 70% had only 1–2 positive sentinel nodes and could avoid ALND [29]. Furthermore, TADPOLE, which will open to recruitment this year, is investigating omission of axillary radiotherapy by comparing the outcomes of TAD alone versus ALND in patients with low-volume nodal disease in the primary setting [26]. These trials, if successful, would address the controversial matter of axillary de-escalation in patients with cN + disease having primary surgery.

## Estimating axillary nodal burden

One of the limitations of the ACOSOG Z011 trial is that it did not obtain preoperative axillary ultrasound for axillary nodal staging and relied on physical examination alone. However, metastatic lymph nodes are not often palpable and are identified after axillary ultrasound and subsequent biopsy of suspicious nodes [10]. Consequently, it is likely that a proportion of patients included in the ACOSOG Z0011 trial were cN0 by physical examination but possibly cN + if they underwent preoperative axillary ultrasound. For that reason, the NCCN guidelines allow SLNB if only 1 or 2 suspicious nodes are seen on ultrasound. In clinical practice, axillary ultrasound is an important imaging modality to assess the axillary lymph node status and is routinely used for all patients with a new breast cancer diagnosis. One of the criticisms of routine use of preoperative axillary ultrasound is that it leads to higher rates of ALND, as abnormal nodes on ultrasound are not reliable indicators of the extent of axillary surgery [27]. Notably, ALND will still be recommended in those with more than 2 positive nodes identified on preoperative imaging.

Physical examination and preoperative imaging lack accuracy in evaluating the burden of disease in the axilla. The accuracy of ultrasound in the diagnosis of axillary nodal metastasis among cN0 patients ranges between 26 and 87% and the sensitivity is improved when coupled with ultrasound-guided needle biopsy [18]. Studies have reported that patients with more than 1 abnormal lymph node identified by ultrasound or MRI were more likely to have more than 3 positive nodes on final pathology compared to patients with only 1 abnormal lymph node on axillary imaging [10, 27]. A meta-analysis found that approximately two-thirds of patients with only 1 suspicious lymph node on preoperative imaging had 1 or 2 metastatic nodes on final pathology and were overtreated by ALND [18]. The correlation between the number of abnormal nodes on preoperative imaging and axillary nodal burden was strongest when 2 or more abnormal nodes were identified on PET/CT scan [28]. However, we do not advocate routine use of PET/CT in the staging of early-stage breast cancer. In addition, large tumours and lobular histology are predictive of higher nodal stage, and de-escalation of axillary surgery may not be applicable in all cases of early-stage cN + breast cancer having primary surgery [36].

Radiologists are also faced with the difficulty of differentiating between 2 and 3 involved nodes, creating a dilemma of whether patients with cN + disease undergoing primary surgery should be managed by SLNB or ALND [17]. By using 1 suspicious lymph node as the cut-off, there is moderate sensitivity and specificity for the prediction of axillary nodal burden at 66% and 73%, respectively [18]. In addition, the MUTAS trial found that FNR of SLNB in patients with cN + disease is reduced to 15% in patients with a single suspicious node on ultrasound [31]. For these reasons, we suggest that the NCCN guidelines be revised to support de-escalation of the extent of axillary surgery at the time of primary surgery in patients with only 1 suspicious or biopsy-proven lymph node in order to satisfy the criteria for omission of ALND.

## Recommendations

We recommend that all patients with a new breast cancer diagnosis undergo preoperative axillary staging by ultrasound. Needle biopsy of abnormal or suspicious axillary lymph nodes should be performed. All patients with cN+ axilla should have staging CT to assess the axillary nodal burden and exclude distant metastases. In patients with HER2-positive or TNBC, a positive axillary lymph node biopsy would triage patients to NACT followed by de-escalation of axillary management depending on their response. Primary surgery should not be performed unless these patients have contraindications to NACT.

**Fig. 1:**
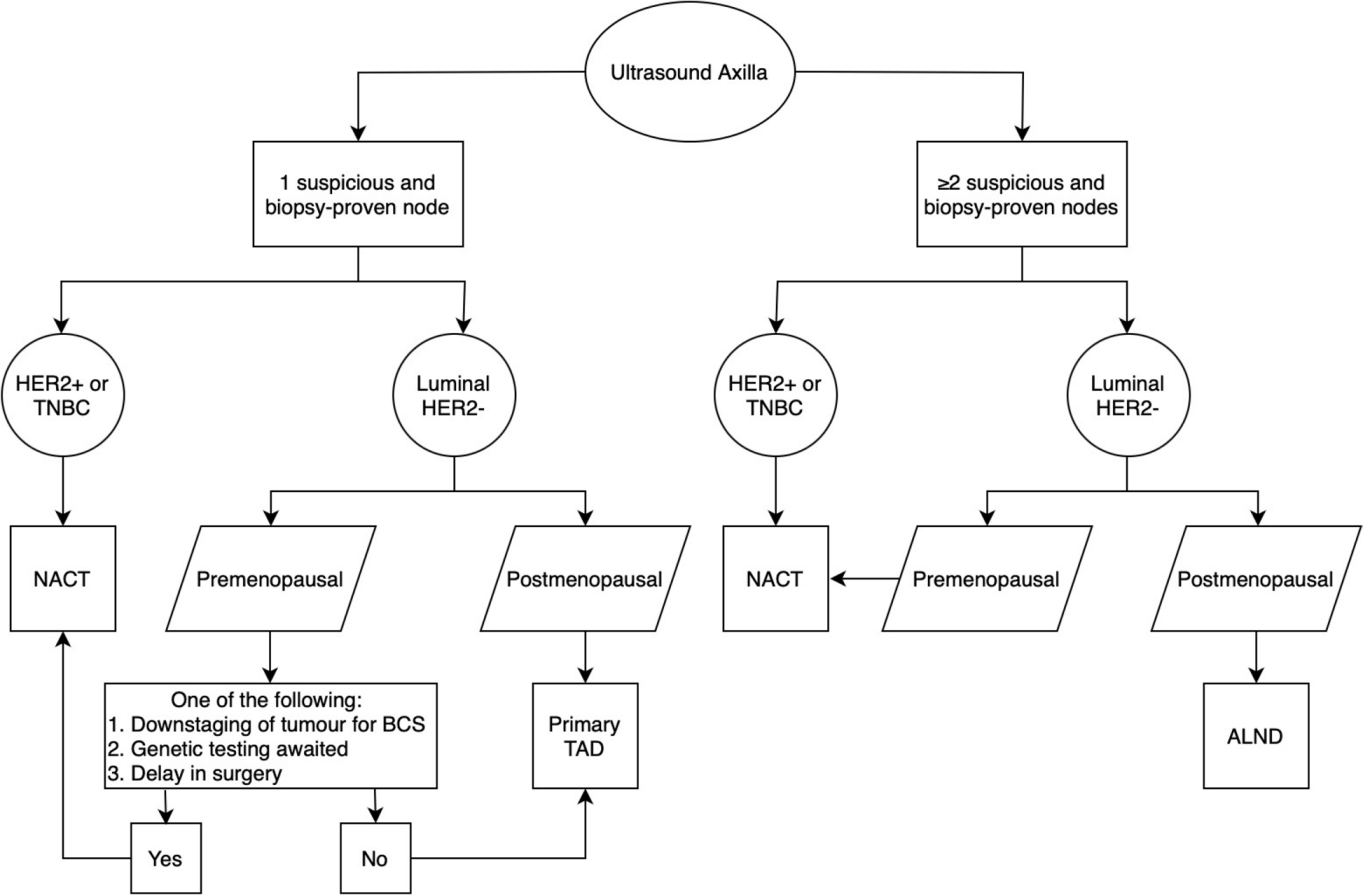
Suggested management of low-volume axilla for patients with cT1-2N1 breast cancer

Conversely, the axillary management of patients with HR-positive and HER2-negative breast cancer with cN + disease depends on their menopausal status and axillary nodal burden on ultrasound. In postmenopausal patients with a single abnormal or suspicious lymph node confirmed positive by needle biopsy, we propose clipping of this solitary lymph node followed by primary surgery in the form of SLNB together with excision of the marked lymph node, according to the NCCN guidelines. If downstaging of the breast tumour is required to facilitate BCS or surgery will be delayed while awaiting genetic testing results or considering reconstructive options, NACT should be given, particularly in premenopausal patients. If NACT will not change the extent of surgical treatment of the breast, premenopausal patients could also be managed by primary TAD. NACT for axillary downstaging alone should not be warranted in these patients. However, if preoperative ultrasound shows multiple abnormal axillary lymph nodes, the probability of extensive nodal involvement is high, and these patients should have NACT accordingly. ALND may be may be justified in postmenopausal patients with 2 abnormal lymph nodes on preoperative imaging when primary surgery is considered, potentially allowing adjuvant chemotherapy decisions to be made by Oncotype DX. Intraoperative frozen sections may be considered in patients undergoing primary surgery with TAD to obviate the need for a second surgery for completion ALND. All patients with cN + disease undergoing primary TAD and no ALND should have axillary radiotherapy (Fig. 1).

## Conclusion

There is a subset of patients with cN + disease undergoing primary surgery who can undergo SLNB or TAD. Identification of a positive node by preoperative imaging and biopsy may triage HER2-positive and TNBC patients to NACT, but it does not represent an indication for ALND in most HR-positive/HER2-negative patients with 1 abnormal node. While current evidence suggests this may be a safe approach, there is lack of prospective data. Long-term outcomes from ongoing trials are eagerly awaited to provide a consensus in this era of de-escalation of axillary treatment. We advocate that NCCN guidelines be revised to support a more oncologically safe approach to surgical axillary staging in patients with cN + disease undergoing primary surgery.

## Data Availability

No datasets were generated or analysed during the current study.
